# Effectiveness of exercise on fatigue in hemodialysis patients: a randomized controlled trial

**DOI:** 10.1186/s13102-020-00165-0

**Published:** 2020-03-18

**Authors:** Farzaneh Salehi, Mahlagha Dehghan, Parvin Mangolian Shahrbabaki, Mohammad Reza Ebadzadeh

**Affiliations:** 1 Clinical Research Unit, Shafa Hospital, Kerman University of Medical Sciences, Kerman, Iran; 2 Nursing Research Center, Kerman University of Medical Sciences, Kerman, Iran; 3 Nursing Research Center, Razi Faculty of Nursing and Midwifery, Kerman University of Medical Sciences, Kerman, Iran; 4 Department of Urology, Bahonar Hospital, Kerman University of Medical Sciences, Kerman, Iran

**Keywords:** Fatigue, Hemodialysis, Exercise, Cycling

## Abstract

**Background:**

Hemodialysis is one of the common therapies in patients with end-stage renal disease. Even patients who receive regular treatment suffer from fatigue, which is one of the main factors leading to poor quality of life. This study aimed to determine the effectiveness of exercising on mini-bikes on fatigue in hemodialysis patients.

**Methods:**

This study is a randomized controlled clinical trial. Thirty-seven hemodialysis patients participated in the study. The patients were randomly allocated to either the intervention group (*n* = 20) or the control group (*n* = 17). The participants in the intervention group exercised on mini-bikes for 20 min twice a week for 3 months. The patients’ fatigue was measured four times during and after the intervention. Multidimensional Fatigue Inventory was used to measure the fatigue level. The total score in the MFI is 4 to 20 for each domain, with the resulting total fatigue score ranging from 20 to 100; thus, the higher the score, the higher the level of fatigue. Data were analyzed by SPSS 18. The repeated measures ANOVA was used to compare the fatigue scores within each group and between the groups at different times.

**Results:**

The mean score of fatigue in the intervention group at the beginning was 58.80 ± 15.29, which steadily decreased to 58.78 ± 13.54, 58.75 ± 14.73, 54.20 ± 15.16, and 54.23 ± 13.60 for the 3 months of intervention and 1 month post-intervention, respectively. In contrast, in the control group, this score was 62.53 ± 16.32 in the beginning, increasing to 64.03 ± 13.91, 64.22 ± 13.07, 69.53 ± 9.22, for the 3 months of intervention and 70.34 ± 7.69 one-month post-intervention. There were significant differences between the intervention group and the control group in the third month (*P* = 0.001) and 1 month after the intervention (*P* < 0.001).

**Conclusions:**

The results showed that rehabilitation through exercising using mini-bikes had a significant impact on preventing further fatigue build-up in hemodialysis patients, making the mini-bike an effective non-pharmaceutical intervention preventing the increase in fatigue experienced by patients undergoing hemodialysis.

**Trial registration:**

Iranian Registry of Clinical Trials: IRCT20180314039100N1. Registered 10 June 2018.

## Background

The annual 5–6% growth in the number of chronic kidney-failure patients makes this disease one of the most important problems in every country, and hemodialysis is the most prominent method for treating patients suffering from it [1]. Hemodialysis patients are constantly dealing with numerous problems such as fatigue due to the chronic nature and the side effects of hemodialysis, which negatively affects the quality of their lives [2]. Fatigue is a subjective sense of weakness, loss of energy, tiredness, and malaise. It is known as a biological warning when human health is at risk. This disorder reduces the sense of well-being and has numerous effects on the physical, emotional, and cognitive dimensions of patient experience [3, 4].

Fatigue reduces self-care activities, disrupts familial and social roles, and decreases the ability to perform routine activities and can lead to unemployment and increased dependence on health care, negatively affecting patient quality of life and self-confidence [5, 6]. 60 to 97% of hemodialysis patients experience some level of fatigue compared to patients with normal kidney function [7]. Some factors leading to fatigue in hemodialysis patients include uremia, anemia, sleep disorders, and psychosocial distress, many of which may be amenable to intervention.

Despite the availability of pharmaceutical interventions such as L-carnitine, used for alleviating fatigue, side effects such as gastrointestinal disorders, nausea, vomiting, stomach upset, heartburn, diarrhea, and sleep disorders as well as drug interactions due to reduced renal excretion and increased drug toxicity have led to a greater tendency to use complementary therapies [8].

One of the important strategies for controlling some of these side effects is exercise [9]. Several studies have confirmed the positive effects of physical activities on different populations [10]. These effects include reduced cardiovascular disease, reduced mortality rates, improvement in and control of blood pressure, controlled blood glucose, increased sense of wellbeing, and improvement in physical performance [9, 11, 12].

Arian and Mortazavi [13] demonstrated that 56% of hemodialysis patients exercised less than once a week, 75% had severe limitations in doing heavy exercises, and 42% had average limitations in their daily activities. This could be due to anemia, blood circulation disorders in extremities, reduced heart performance, and decreased daily physical activity. Other important factors such as anxiety, depression, spending more time sleeping, and inactivity further limit the physical capacity of those undergoing dialysis [14]. Also, the lack of motivation and developing too many medical complications have been associated with a lower physical capacity in hemodialysis patients [15]. It seems that control of fatigue in hemodialysis patients can lead to physical betterment as well as psychological improvements such as reduction of depression and anxiety, improvement in self-care, reduction of dependence, and improvement in quality of life, the sense of wellbeing, and increased sleep quality in patients [16–20].

Muscle and joint stretching may be difficult for the elderly and those with bone problems. Also, the fistula in the forearm and being attached to a hemodialysis machine are serious barriers to performing stretching exercises, making it impossible to use this intervention during hemodialysis. Therefore, in order to overcome the limitations of the physical activity for patients under hemodialysis, we opted for the use of “mini-bikes”, which are more affordable and accessible than other equipment and have far fewer ergonomic risks. A mini bike is much more suitable than a regular bike for exercise patients undergoing hemodialysis because it can easily be used for patients who have to remain in bed for as long as 3 h. Minibikes can also benefit older patients and those with bone problems when used inactively. Such easily executable exercise can relieve the patient from the three- to four-hour inactivity during hemodialysis.

Due to the importance of fatigue, which is a neglected aspect of the treatment process of hemodialysis patients, and the emerging data showing that exercise is able to alleviate fatigue, this study aimed to assess the effect of exercising on mini-bikes on fatigue in hemodialysis patients, with the hypothesis that the use of mini-bikes could affect fatigue in patients undergoing hemodialysis.

## Methods

### Study design and setting

The present study was a randomized controlled clinical trial conducted in the hemodialysis units of Shafa Hospital and Jawad Al Aemeh Center, affiliated with Kerman University of Medical Sciences.

### Sample size and sampling

As demonstrated in a previous study, where researchers examined the effect of an exercise program on reducing fatigue and improving sleep in long-term hemodialysis patients, the present study (16 interventions and 16 controls) had sufficient power (80%) to detect a mean difference of fatigue score of 3.1 (Fatigue score: μ_1_ = 7.6, μ_2_ = 10.7, S_1_ = 3.3, and S_2_ = 2.9) [20].

**Fig. 1:**
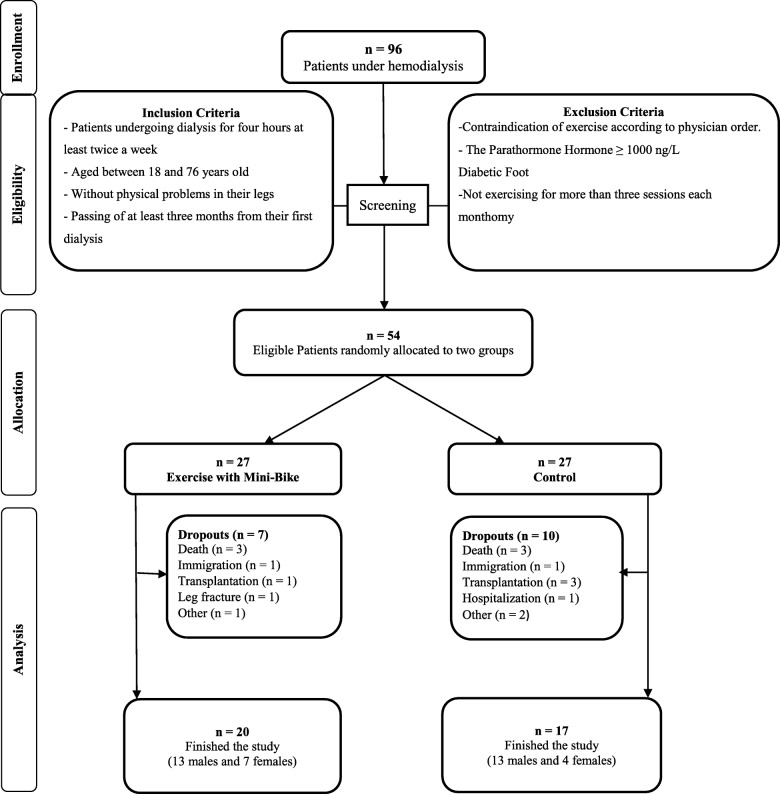
Explanation of sample size and sampling

The researcher selected eligible people after coordinating with the head of the hospital and the dialysis department. The inclusion and exclusion criteria are presented in Fig. 1. A total of 96 patients under hemodialysis were studied using the census method. Fifty-four patients were eligible to enter the study. The first researcher assigned twenty-seven patients to the intervention and control groups using the minimization method. First, the sex and age of the two groups were matched (± 2), and they were then randomly allocated to one of the groups by throwing dice. Finally, 20 patients in the intervention group and 17 patients in the control group finished the study (Fig. 1).

### Measurement

Demographic questionnaire and Multidimensional Fatigue Inventory (MFI) were used to collect information. The demographic questionnaire included questions on age, gender, education, income, the number of children, exercise, duration of dialysis, cause of dialysis, transplant rejection, and other diseases.

The MFI, used to measure the level of fatigue, included 20 items and 5 subscales of fatigue (4 questions), physical fatigue (4 questions), decline in activity (4 questions), decline in motivation (4 questions), and mental fatigue (4 questions). Scoring was done using a Likert 5-point scale (1 = Yes, entirely correct to 5 = No, entirely wrong). The total score was 4–20 for each domain, leading to a total fatigue score ranging from 20 to 100. The higher the score, the higher the level of fatigue [21]. The validity and reliability of the questionnaire have been confirmed in the Iranian population [22]. Many researchers have used this instrument to measure fatigue [23–25].

### Data collection and intervention

After getting to know the patients undergoing hemodialysis the day before the study, a nephrologist examined the patients to make sure there was no medical restriction for entering the study. Afterwards, the MFI was completed by the intervention and control groups. Then, the exercise program was conducted twice a week for 12 weeks simultaneously with the hemodialysis sessions. Patients exercised for 20 min during the first 2 h of the hemodialysis using the electric exercise bike (Model number: TD001P-1-A#5815, Brand name: TODO, Origin: China) (Additional file 1).

Since patients experience fluid accumulation in the body before hemodialysis begins, such excess fluids must be removed within 4–5 h. This rapid release of fluids from the body can sometimes lead to hemodynamic changes and symptoms such as hypotension or cramps [26]. Therefore, based on the researcher’s clinical experience, physician recommendations, and literature review, the best time for intervention is 30 min after the initiation and during the first 2 h of dialysis [27]. The researcher placed the bike on the bed, fixed the patient’s feet to the pedals using adhesive straps, and knee range of motion was determined for each subject. Patients performed passive pedaling at low power for 20 min at a speed of 30 rpm during the first 2 h of every dialysis session. Participants were instructed on how to exercise and verbal encouragement was provided to them during exercise. Before, during, and after exercise, clinical assessment, blood pressure, heart rate and body temperature were obtained. If the participant had a blood pressure of 180/110 mmHg and higher, systolic pressure lower than 90 mmHg, chest pain, shortness of breath, or high body temperature (> 37.8oC) before or during dialysis, the exercise would be discontinued. None of the patients suffered from such complications and all participated without interruption.

The two groups filled the MFI at the end of each month (1–3, and) during the intervention and 1 month after the intervention. Given the assumption that continuous, long-term exercise can affect the level of fatigue, the study attempted to measure the effect of long-term exercise on fatigue (3 months) as well as the level of fatigue at different times (1 month, 2 months, 3 months, and 1 month after intervention). The study lasted from 15 October 2018 to 22 April 2019.

### Data analysis

Data were analyzed by SPSS 18. Descriptive statistics were used to describe the demographic and clinical characteristics of the participants and other variables of the study. The independent t-test, chi-squared test, and Fisher’s test were used to compare the demographic and clinical characteristics of the samples between the two intervention and control groups. Since the parametric conditions were confirmed, the repeated measures ANOVA was used to compare the fatigue scores within each group and between the groups at different times. A *P*-Value below 0.05 was considered significant. There were some instances of missing data in relation to some questions in various measurements of the fatigue scale. As the fatigue scores had normal distribution at all times in both groups, the missing values were replaced with the mean in each group.

## Results

The mean ages were 57.8 ± 9.17 yrs. and 54.65 ± 10.02 yrs. in the intervention and control groups, respectively. There were no significant differences between the two groups in age, gender, education, the number of children, income, exercise, history of dialysis, history of transplant rejection, cause of disease, and history of other diseases (*P* > 0.05) (Table 1).

**Table 1:** Comparison of patients’ characteristics between the intervention and control groups

Group		The control group		The intervention group		Statistical test	*P* value
Variable		Mean	SD	Mean	SD		
Age (yr.)		54.65	10.02	57.80	9.17	0.99^a^	0.32
Duration of dialysis (month)		36.94	20.37	43.5	39.19	- 0.21^b^	0.84
		n	%	n	%		
Sex	Female	4	23.5	7	35	0.58^c^	0.45
	Male	13	76.5	13	65		
Education	Illiterate	6	35.3	2	10	5.6^d^	0.24
	Under diploma	4	23.5	11	55		
	Diploma	4	23.5	3	15		
	Academic	3	17.7	4	20		
Doing exercise	Regularly	0	0	3	15	2.89^d^	0.32
	Sometimes	8	47.1	6	30		
	Never	9	52.9	11	55		
Cause of kidney failure	Diabetes	10	58.8	12	60	2.7^d^	0.75
	Hypertension	3	17.6	4	20		
	Polycystic kidney	2	11.8	4	20		
	Others	2	11.8	0	0		
History of other disease	Yes	4	23.5	11	55	3.78^c^	0.053
	No	13	76.5	9	45		

^a^ Independent T Test

^b^ Mann Whitney U Test

^c^ Chi-squared Test

^d^ Fisher exact test

The mean total score of fatigue in the experimental group was 58.80 ± 15.29 in the beginning, and continuously decreasing in the first month (58.78 ± 13.54), second month (58.75 ± 14.73), third month (54.20 ± 15.16), and 1 month post- intervention (54.23 ± 13.60). However, there were no significant differences within groups at the various time points (*P* = 0.12). The fatigue score in the control group was 64.53 ± 16.32 in the beginning, and it steadily increased in the first month (64.03 ± 13.91), second month (64.22 ± 13.07), third month (69.53 ± 9.22), and 1 month after the intervention (70.34 ± 7.69). However, there were no significant differences within groups at the various time points (*P* = 0.06). The total fatigue score significantly decreased in the intervention group compared with the control group in the third month (*P* = 0.001), and 1 month after the intervention (*P* < 0.001).

**Fig. 2:**
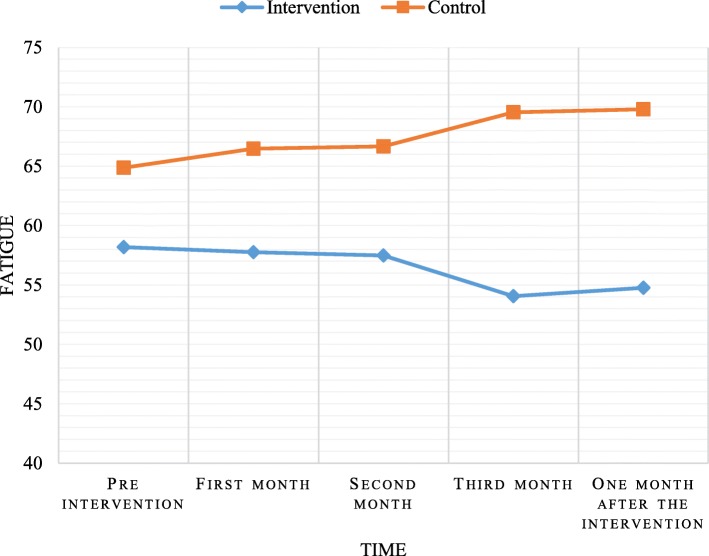
Comparison of mean fatigue at different times between intervention and control groups

Based on these results, during the intervention fatigue was slightly better, although not statistically significant, and were precluded from the progression of fatigue (Table 2, Fig. 2). Results of this study showed that there was a significant difference between the experimental and control groups in subscales of fatigue such as a decline in motivation (*P* = 0.02) and mental fatigue (*P* = 0.046). Also, there was a significant difference between the experimental and control groups in all subscales of fatigue during the third month of intervention and 1 month after intervention (Table 3). In addition, the results showed that exercise prevented the development of mental fatigue in the experimental group.

**Table 2:** Comparison of the fatigue scores at different times between the intervention and the control groups

Group	The control group		The intervention group		Statistical test^a^	*P* value	Partial Eta Squared	Mean Difference	*P* value^b^
Fatigue score	Mean	SD	Mean	SD					
Pre intervention	62.53	16.32	58.80	15.29	5.72	0.02	0.14	−3.73	0.48
First month	64.03	13.91	58.78	13.54				−5.25	0.25
Second month	64.22	13.07	58.75	14.73				−5.47	0.24
Third month	69.53	9.22	54.20	15.16				−15.33	0.001
One month after the intervention	70.34	7.69	54.23	13.60				−16.12	< 0.001
Greenhouse-Geisser	3.12		2.25						
*P* value	0.06		0.12						

^a^ Repeated measured ANOVA

^b^ Adjustment for multiple comparisons: Bonferroni

**Table 3:** Comparison of the fatigue dimensions’ scores at different times between the intervention and the ontrol groups

Group		The control group		The intervention group		Statistical test^a^	*P* value	Partial Eta Squared	Mean Difference	*P* value^b^
Fatigue dimensions		Mean	SD	Mean	SD					
General Fatigue	Pre intervention	12.24	3.94	12.60	3.44	1.88	0.18	0.05	0.36	0.77
	First month	12.94	3.53	12.72	3.37				−0.22	0.84
	Second month	12.88	3.5	12.61	3.20				−0.26	0.80
	Third month	13.93	3.10	10.95	5.17				−2.98	0.04
	One month after the intervention	14.19	2.74	10.83	4.38				−3.36	0.007
	Greenhouse-Geisser	4.36		2.08						
	*P* value	0.01		0.14						
Physical Fatigue	Pre intervention	14.53	4.16	13.75	4.69	2.81	0.10	0.07	−0.78	0.60
	First month	13.69	2.87	13.39	3.68				−0.30	0.78
	Second month	14.44	3.12	13.5	3.96				−0.94	0.41
	Third month	15.67	2.13	12.21	3.54				−3.46	0.001
	One month after the intervention	15.75	1.95	12.78	4.22				−2.97	0.008
	Greenhouse-Geisser	3.33		2.62						
	*P* value	0.04		0.07						
Decline in activity	Pre intervention	14.71	4.97	12.95	5.42	3.94	0.05	0.10	−1.76	0.32
	First month	14.25	3.97	11.83	4.63				−2.42	0.09
	Second month	15.06	4.19	12.50	5.38				−2.56	0.10
	Third month	15.73	2.66	13.16	5.10				−2.57	0.06
	One month after the intervention	15.94	2.69	13.00	5.06				−2.94	0.03
	Greenhouse-Geisser	1.45		0.71						
	*P* value	0.25		0.52						
Decline in Motivation	Pre intervention	10.71	4.00	10.30	3.58	6.11	0.02	0.15	−0.41	0.75
	First month	11.62	3.03	10.28	3.56				−1.35	0.21
	Second month	11.25	3.59	9.94	3.15				− 1.31	0.23
	Third month	12.73	3.22	9.26	3.44				−3.47	0.002
	One month after the intervention	12.44	3.52	9.11	3.06				−3.33	0.003
	Greenhouse-Geisser	1.83		0.99						
	*P* value	0.18		0.38						
Mental Fatigue	Pre intervention	10.35	4.43	9.2	3.05	4.26	0.046	0.11	−1.15	0.36
	First month	11.38	3.77	10.67	3.11				−0.71	0.52
	Second month	10.44	4.15	10.33	3.72				−0.10	0.93
	Third month	11.47	3.2	8.63	2.75				−2.84	0.004
	One month after the intervention	12.06	3.71	8.44	2.28				−3.62	0.001
	Greenhouse-Geisser	1.42		3.30						
	*P* value	0.25		0.04						

^a^ Repeated measured ANOVA

^b^ Adjustment for multiple comparisons: Bonferroni

## Discussion

The present study showed that exercise with mini bikes prevented increase in fatigue and its subscales, especially mental fatigue, in the test group. On the other hand, the control group experienced a progressive increase in their fatigue levels, which amounted to a statistically significant level in later months. This intervention supports the study hypothesis that exercise with mini bikes can affect fatigue among hemodialysis patients. The results of this study are important because, on the one hand, patients are increasingly showing interest in non-pharmaceutical methods, and on the other, specialists refuse to include non-pharmaceutical methods that are not scientifically confirmed in their intervention regimens.

The results of the present study showed that most of the participants in the two groups suffered from fatigue before the intervention. Jablonski reported that 69 to 77% of hemodialysis patients suffered from fatigue and were in need of non-pharmaceutical interventions. According to the study of Lashkari et al., in Iran, 42, 36, and 19% of hemodialysis patients suffered from high, medium, and low levels of fatigue, respectively [28]. Mohamed et al.reported that the majority of hemodialysis patients in Egypt experienced fatigue [29]. Jhamb et al. noted a 60–97% prevalence of fatigue in Chinese patients undergoing hemodialysis [30]. Picariello et al., showed that fatigue was common in hemodialysis patients in the UK, and that there was a strong correlation between the mental and physical factors of fatigue [31].

The results of this study showed that there was a significant difference in the levels of fatigue between the experimental and control groups in the second and third months of intervention and 1 month after the intervention. Another notable point was that the level of fatigue increased in the control group over time. It can be deduced from the results of this study that exercise is a particularly promising way to curb the natural progression of fatigue that hemodialysis patients experience through time; the fatigue in patients under hemodialysis will increase if it is not appropriately addressed. In line with our results, Riahi et al. [32], reported a decrease in fatigue and an increase in physical strength among hemodialysis patients after 5 months of exercising on a mini-bike for 60 min three times a week. According to research by Macdonald et al. [33], exercising three times a week with a suitable bicycle could lead to increased muscle mass in end-stage renal disease (ESRD) patients through adjustment of the insulin-like growth factor. Researchers found that attention to preventing mental fatigue is important because mental fatigue causes symptoms such as lack of motivation, irritability, loss of appetite, and insomnia. Mental fatigue can affect patient quality of life both in the short and in the long term [34]. Similar results were reported by Maniam et al. [20], Soliman et al. [35], Yutkuran et al. [36], Malagoni et al. [37], and Liu et al. [38]. However, these authors employed different kinds of exercise programs such as stretching, intradialytic range of motion exercise programs, yoga-based exercises, walking programs, and aerobic exercises. Maniam et al. [20] showed that 15 min of stretching before hemodialysis three times a week for 12 weeks could decrease fatigue and improve sleep disorders in patients. According to results of Soliman et al. [35], a significant reduction was seen in the level of fatigue and electrolytes such as serum phosphate, potassium, calcium, urea, and creatinine among ESRD patients undergoing Hemodialysis after an 8-week intradialytic range of motion exercise program. Also, fatigue and musculoskeletal problems were reported to improve with yoga [36]. In addition, an original 6-month walking program was effective on post-dialysis fatigue, physical capacity, and health-related quality of life among hemodialysis patients [37]. The results of Liu et al. [38] suggest that aerobic exercise plays an important role in physical function and decreases depression. Motedayen et al. [39] and Tayyebi et al. [40] showed that body-mind intervention is effective on fatigue and dialysis adequacy. It is suggested that exercise combined with mental training can have a positive effect on treatment outcomes. Several studies have demonstrated that exercise during dialysis not only decreases fatigue, but also increases self-confidence and self-efficacy [27, 32, 39]. A systematic meta-analysis study showed that exercise training helps hemodialysis patients to reduce the severity of restless leg syndrome, depression, and fatigue [41].

Researchers believe that exercise widens muscular arteries, improves perfusion, and alleviates diseases affecting blood circulation in muscles [42]. Therefore, due to the improvement in perfusion and enhanced blood circulation and better toxin elimination and higher muscular strength, and consequently, lower levels of fatigue and impotence, we can expect improvement in the quality of life of patients undergoing hemodialysis. This study provides a more time-efficient option for exercise among hemodialysis patients as the method used did not necessitate extra time, costs, and transportation. Therefore, it seems that mini-bike exercise is safe and effective in the rehabilitation and complementary treatment of patients undergoing hemodialysis. Another point worth noticing is that the benefits of preventing the progression of fatigue remained at least 1 month after the discontinuation of the exercises. It is concluded that long-term exercise can have a significant impact on preventing long-term fatigue. Researchers believe that an intervention is valuable when its effects are sustained over time [43].

This study had some limitations. Firstly, the knowledge, experience, emotional and mental characteristics, and cultural backgrounds of the patients affected their level of motivation and desire. Moreover, although two main centers were selected, and all eligible patients were included, the sample size decreased due to the dropout. It is recommended that this study be conducted with a larger sample size. Another limitation of this study was that we did not make any objective measurements of fatigue. It is suggested that this issue be considered in future research.

## Conclusions

Altogether, the present study showed that exercising was effective in prevention of progression of fatigue in patients under hemodialysis over time, at least up to 1 month after the intervention. Therefore, as paying attention to hemodialysis patients’ quality of life is an important goal of dialysis centers, we can recommend the use of this method to prevent the progression of fatigue in them. According to the experiences of researchers during the study and observing the patients’ enthusiasm for doing the exercises, it seems that we need to use these kinds of interventions as non-pharmaceutical therapies combined with other treatments for patients under hemodialysis. Of course, we need to realize that doing these kinds of activities requires informing the health care team members such as the physicians, nurses, and other members about the advantages and the correct way of implementing the exercise program. Finally, it is recommended that other studies focusing on comparing the effectiveness of exercise therapy and pharmaceutical therapy on fatigue in hemodialysis patients be conducted to provide better service to the patients. It should also be pointed out that further research is required to determine the optimal exercise prescription guidelines for hemodialysis patients.

## Supplementary information

**Additional file 1.** Is a photo of the mini bike was used in this study. This photgraph was taken by the authors.

## Data Availability

The datasets used and/or analyzed during the current study are available from the corresponding author on reasonable request.

## Abbreviations

ESRD: End-Stage Renal Disease
MFI: Multidimensional Fatigue Inventory
