# Supplementary figures and images for: Effectiveness of exercise on fatigue in hemodialysis patients: a randomized controlled trial
Source: BMC Sports Sci Med Rehabil. 2020 Mar 18;12:19. doi: 10.1186/s13102-020-00165-0 (PMC7081561; doi:10.1186/s13102-020-00165-0)

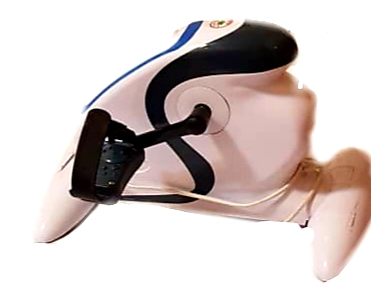


The mini bike was used in the study

Supplement: Supplementary file 1 — Additional file 1. Is a photo of the mini bike was used in this study. This photgraph was taken by the authors. [file 13102_2020_165_MOESM1_ESM.docx]
